# Association of *SOX2* and *Nestin* DNA amplification and protein expression with clinical features and overall survival in non-small cell lung cancer: A systematic review and meta-analysis

**DOI:** 10.18632/oncotarget.9145

**Published:** 2016-05-02

**Authors:** Qingbao Li, Fang Liu, Yuan Zhang, Lei Fu, Cong Wang, Xuan Chen, Shanghui Guan, Xiangjiao Meng

**Affiliations:** ^1^ Department of Cardiac Surgery, Shandong Provincial Hospital Affiliated to Shandong University, Jinan, 250021, China; ^2^ Department of Image, Shandong Medical College, Jinan, 250002, China; ^3^ Department of Laboratory Medicine, Shandong Medical College, Jinan, 250002, China; ^4^ Department of Radiation Oncology, Shandong Cancer Hospital and Institute, Jinan, 250117, China; ^5^ Department of Radiation Oncology, Qilu Hospital of Shandong University, Jinan, 250012, China

**Keywords:** meta-analysis, SOX2, Nestin, clinical outcome, non-small cell lung cancer

## Abstract

Up to now, the prognosis of non-small cell lung cancer (NSCLC) is poor. With progress of cancer biology, a number of genes have been investigated for predicting prognosis of NSCLC, such as cancer stem cell markers SRY (sex determining region Y)-box 2 (*SOX2*) and Nestin. Recently, a series of studies have been performed to examine the associations of *SOX2* and Nestin with clinical parameters and prognosis in NSCLC, however, the results were not consistent. In the present study, we conducted a systematic review and meta-analysis to summarize the associations. Four English databases (PubMed, ISI web of science, Embase, and Ovid) were used to search the relevant studies with the last date of November 10, 2015. The pooling analyses were stratified by DNA amplification and protein expression. The pooling ORs or HRs were used to assess the strength of the associations. Finally, we included 19 articles for *SOX2* and six articles for *Nestin* according to the inclusion and exclusion criteria. The pooling analyses revealed that there were significant associations between *SOX2* DNA amplification and clinical features of NSCLC, gender, smoking status, squamous cell cancer (SCC) histology, and differentiations. And significant associations were also identified between *SOX2* protein expression and clinical parameters, smoking status and SCC histology. For Nestin, its protein expression was correlated with lymph node metastasis and stage. Simultaneously, we found that high/positive SOX2 alterations, either DNA amplification or protein expression, were favorable for overall survival (OS) in NSCLC. On the contrary, high/positive *Nestin* protein expression was poor for OS.

## INTRODUCTION

Lung cancer is the leading cause of cancer death worldwide and its 5-year relative survival rate is low [1]. Traditionally, it is classified into two major subtypes, small cell lung cancer (SCLC) and non-small cell lung cancer (NSCLC). The latter can be subdivided into adenocarcinoma (ADC), squamous cell carcinoma (SCC), and large cell carcinoma (LCC) [2].

In recent years, some activated oncogenes such as Epidermal Growth Factor Receptor (EGFR) mutations and Anaplastic Lymphoma Kinase (ALK) rearrangements have been found and used as novel therapeutic targets [3–5]. All these progresses encourage the researchers to identify new biomarkers or therapeutic targets. Of which, the cancer stem cell markers such as SRY (sex determining region Y)-box 2 (*SOX2*) and *Nestin* have gotten researchers interested.

*SOX2* locates on chromosome 3q26.33 and encodes a transcription factor of 317 amino acids [6, 7]. It has been reported to be involved in pluripotency regulation in embryonic stem cells and the morphogenesis and homoeostasis of tracheobronchial epithelia [8]. Recently, *SOX2* aberrant DNA amplification and protein expression have been found in various types of tumors. Functional experiments suggest that *SOX2* is responsible for cellular proliferation, tumor invasion and migration, self-renewal, maintenance in cancer stem cell populations, and lung tumorigenesis [6, 9–12]. It also has been reported that DNA amplification and protein expression of *SOX2* are associated with clinicopathological features and prognosis in lung cancers, however, the results are not always consistent [13–16]. Although a meta-analysis in the year of 2013 has been performed to summarize the associations, the included studies were relatively rare and the authors do not distinguish *SOX2* DNA amplification, mRNA expression, and protein expression [17].

*Nestin* is a member of the intermediate filament (IF) family and serves as a potential proliferative and muti-potency marker in progenitor and stem cells [18, 19]. *Nestin* has been also found to have an anti-apoptotic function through inhibiting caspase activation [20]. Recent observations have revealed a link between *Nestin* aberrant expression and malignant characteristics and poor prognosis in different cancers [21–25].

In the present study, we performed a systematic review and meta-analysis to investigate the associations of DNA amplification and protein expression of *SOX2* and *Nestin* with clinicopathological features and overall survival in NSCLC.

## RESULTS

### Study characteristics

**Figure 1:**
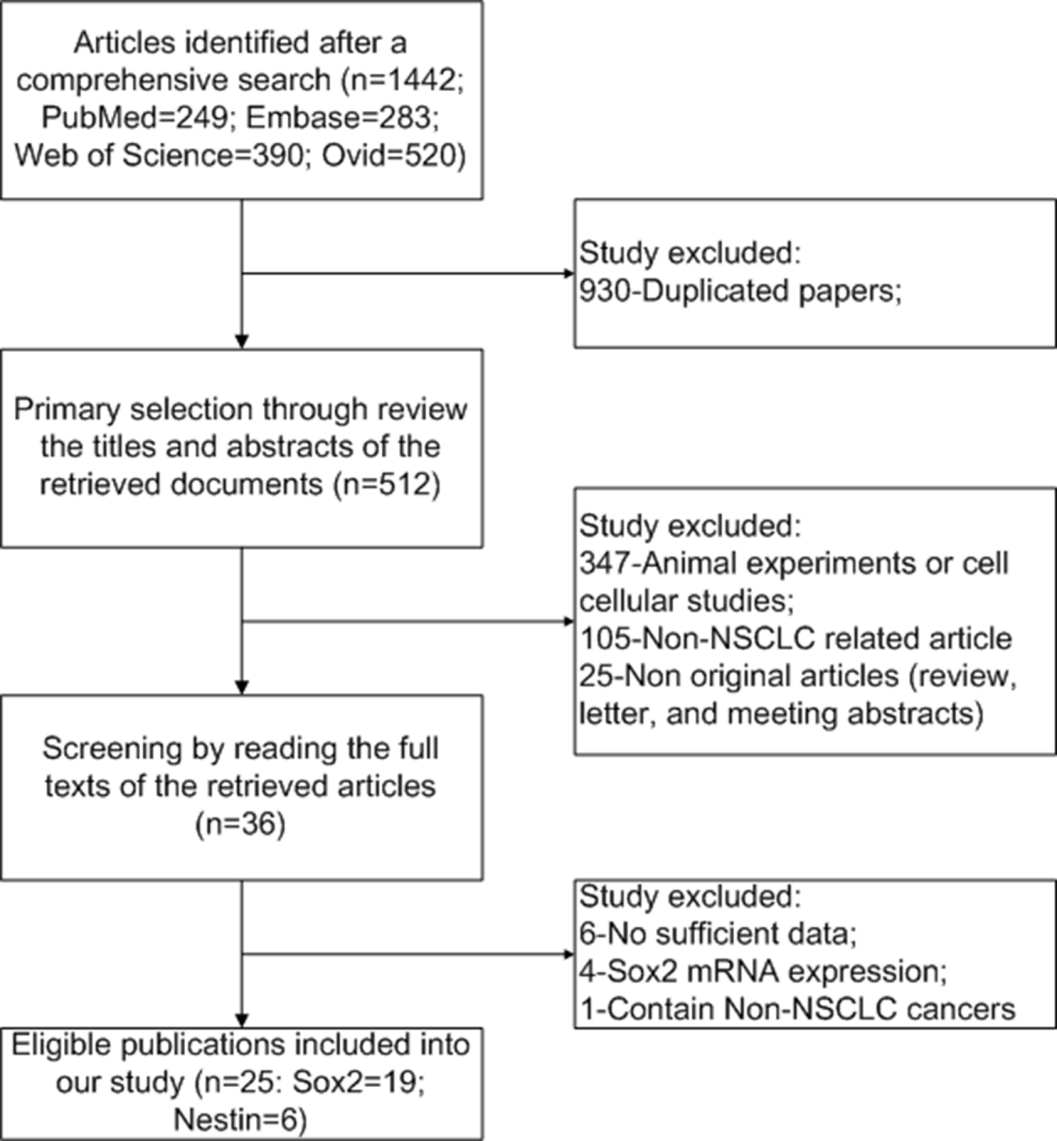
Flow chart of study selection

The literature selection process was shown in Figure 1. Four English databases were used and a total of 1442 documents were initially identified. After excluding those duplicated records, animal experiments or cellular studies, non-NSCLC related studies, and non-original articles, 36 full texts were left for further evaluation. Subsequently, six articles were excluded due to insufficient data [26–31], and one was excluded because it contained other type of lung cancer besides NSCLC [32]. Here, we only focused on DNA amplification and protein expression. Then another four studies were excluded due to only reporting the *SOX2* or *Nestin* mRNA related data [33–36]. Finally, 25 papers were included in the present study [13–16, 21, 37–56]. Of which, 19 articles reported *SOX2* DNA amplification and/or protein expression [13–16, 37–51], six articles reported *Nestin* protein expression, and none reported *Nestin* DNA amplification [21, 52–56]. In addition, Velcheti et al. [46] and Iijima et al. [50] reported two independent cohorts, respectively, and each cohort was considered as independent study in the meta-analysis. The included studies were published from 2010 to 2015 and the sample size ranged from 33 to 758. In the original studies, the DNA amplification was determined by PCR or FISH (*n* = 3 and 6) and the protein expression was determined by IHC or IF (*n* = 19 and 2). The detailed characteristics of the included studies were shown in Table 1.

**Table 1:** Characteristics of the included studies

Reference	Country	Patient No.	Age (year)	Method	Cut-off value	Protein/ Amplification	Positive/ Negative
**SOX2**							
Yuan et al. 2010	USA	57		IHC	SCC (high > 270, low < 140); ADC (high > 193, low < 10)	Protein	37/19
Yuan et al. 2010	USA	57		qPCR	4 copy	Amplification	8/49
Lu et al. 2010	USA	40		IHC	5%	Protein	19/21
Sholl et al. 2010	USA	104	68(36–91)	IHC	5%	Protein	52/52
Sholl et al. 2010	USA	66		IHC	5%	Protein	41/25
Wilbertz et al. 2011	Switzerland/USA	758		FISH	30%	Amplification	224/534
Cai et al. 2011	China	115	58(27–77)	PCR	Ratio > M + 2SD	Amplification	30/85
Cai et al. 2011	China	150	58(27–77)	IHC	5%	Protein	79/71
Koji et al. 2011	Japan	309		IHC	5%	Protein	
Sasaki et al. 2012	Japan	127	66.0 ± 10.2	PCR	4 copy	Amplification	42/85
Brcic et al. 2012	American	147		IHC	5%	Protein	14/52
Brcic et al. 2012	American	147		FISH	CN/chrom > 2	Amplification	18/52
Li et al. 2012	China	44		IHC	10%	Protein	31/13
Chen et al. 2012	China	381		IHC	10%	Protein	374/7
Velcheti et al. 2013	Greek	340	62.32 ± 9.04	IF	Score > 193	Protein	418/229
Velcheti et al. 2013	USA	307	65.17 ± 9.92	IF	Score > 193	Protein	
Chou et al. 2013	China	175		IHC	No stain in nuclear	Protein	51/124
Yusuke et al. 2015	Japan	282	67(33–86)	FISH	Mean value	Amplification	34/244
Toschi et al. 2015	Italy	447		FISH	4 copy or presence of gene cluster	Amplification	105/340
Yoon et al. 2015	Korea	33	66(48–73)	IHC	Internal control	Protein	22/11
Yoon et al. 2015	Korea	33	66(48–73)	FISH	10 green signal	Amplification	26/7
Iijima et al. 2015	China cohort	57		IHC	H-score > 0	Protein	40/17
Iijima et al. 2015	Japan cohort	66		IHC	H-score > 0	Protein	45/21
Zheng et al. 2015	China	162	61.6(40–88)	IHC	100 score	Protein	85/65
Zheng et al. 2015	China	162	61.6(40–88)	FISH	4 gene copy	Amplification	50/61
**Nestin**							
Chen et al. 2010	China	52	58.2 ± 10.0	IHC	8.4(median histoscore of *Nestin*)	Protein	25/27
Janikova et al. 2010	Czech	121		IHC	10%	Protein	74/38
Ryuge et al. 2011	Japan	173	64(34–85)	IHC	5%	Protein	27/144
Skarda et al. 2012	Czech & Israel	115	60.3	IHC	H-score > 0	Protein	40/74
Chen et al. 2014	China	71	57.6 ± 9.8	IHC	8.4(median histoscore of Nestin)	Protein	35/36
Sterlacci et al. 2014	Austria	215		IHC	Median % positive staining cell	Protein	57/269

### Meta-analysis results

### SOX2

**Figure 2:**
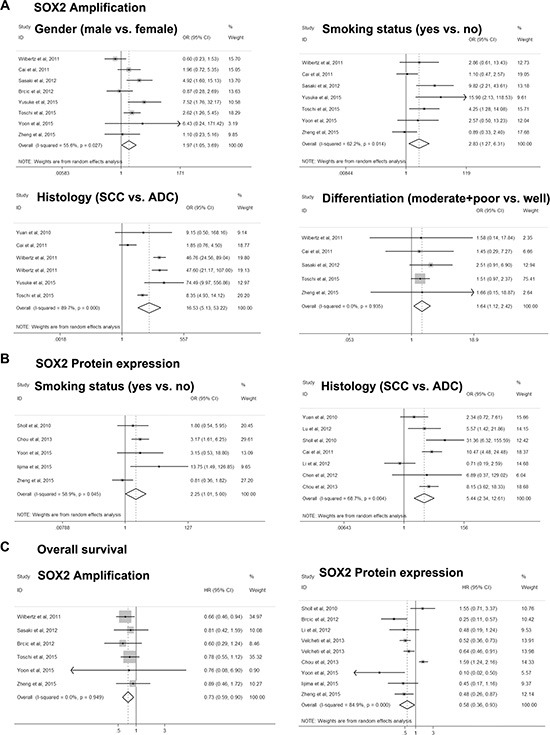
Forest plot for associations of *SOX2* with clinicopathological features and overall survival in NSCLC

Significant associations were identified between high/positive *SOX2* DNA amplification and clinicopathological features, gender (OR = 1.969, 95% CI = 1.050–3.693, *P* = 0.035), smoking status (OR = 2.830, 95% CI = 1.269–6.310, *P* = 0.011), histology (OR = 8.136, 95% CI = 2.136–30.997, *P* = 0.000), differentiation (OR = 1.644, 95% CI = 1.119–2.415, *P* = 0.011), and OS (HR = 0.732, 95% CI = 0.593–0.904, *P* = 0.004) (Figure 2 and Table 2). For *SOX2* protein expression, *i*ts associations with smoking status (OR = 2.245, 95% CI = 1.008– 5.001, *P* = 0.048), histology (OR = 5.437, 95% CI = 2.344– 12.610, *P* = 0.000), and OS (HR = 0.579, 95% CI = 0.359–0.934, *P* = 0.025) were found (Figure 2 and Table 2).

**Table 2:** Meta-analysis results

Clinical parameters	*N*	OR/HR	OR/HR95%CI	*P_OR_*	Model	*I*^2^	*P_hetero_*	*P_Eegg_*	*P_Egger_*
**SOX2 Amplification**									
Gender(male vs. female)	8	**1.969**	**1.050–3.693**	**0.035**	R	**55.6**	**0.027**	0.711	0.652
Age (≤ 60 vs. > 60 or ≤ 65 vs. > 65)	3	0.857	0.507–1.448	0.563	F	0.6	0.365	1.000	0.367
Smoking status (yes vs. no)	7	**2.830**	**1.269–6.310**	**0.011**	R	**62.2**	**0.014**	0.368	0.052
Histology (SCC vs. ADC)	6	**16.530**	**5.134–53.221**	**0.000**	R	**89.7**	**0.000**	0.707	0.885
Differentiation (moderate+poor vs. well)	5	**1.644**	**1.119–2.415**	**0.011**	F	0.0	0.935	0.462	0.629
Differentiation (poor vs. well+moderate)	3	0.807	0.317–2.054	0.654	R	**68.2**	**0.041**	1.000	0.796
Lymph node metastasis (N_0_ vs. N_1–3_)	5	0.943	0.678–1.312	0.728	F	0.0	0.650	0.806	0.688
Lymph nodemetastasis (N_0–1_ vs. N_2–3_)	3	0.903	0.468–1.743	0.761	F	0.0	0.418	0.308	0.168
Stage (I vs. II–IV)	5	1.222	0.860–1.737	0.263	F	0.0	0.855	0.221	0.363
Stage (I–II vs. III–IV)	4	1.226	0.877–1.714	0.232	F	0.0	0.849	0.734	0.690
OS	6	**0.732**	0.593–0.904	**0.004**	F	0.0	0.949	0.707	0.794
**SOX2 Protein expression**									
Gender(male vs. female)	9	1.345	0.726–2.493	0.558	R	**55.1**	**0.023**	0.917	0.738
Age (≤ 60 vs. > 60 or ≤65 vs. > 65)	6	0.439	0.104–1.857	0.263	R	**90.3**	**0.000**	0.368	0.199
Smoking status (yes vs. no)	5	**2.245**	1.008–5.001	**0.048**	R	**58.9**	**0.045**	0.806	0.537
Histology (SCC vs. ADC)	7	**5.437**	2.344–12.610	**0.000**	R	**68.7**	**0.004**	1.000	0.749
Differentiation (moderate+poor vs. well)	6	1.082	0.695–1.685	0.726	F	0.0	0.694	1.000	0.471
Differentiation (poor vs. well+moderate)	9	0.723	0.517–1.011	0.058	F	14.2	0.316	1.000	0.496
Lymph node metastasis (N_0_ vs. N_1-3_)	3	1.078	0.649–1.789	0.772	F	0.0	0.693	1.000	0.952
Lymph nodemetastasis (N_0-1_ vs. N_2-3_)	1								
Stage (I vs. II–IV)	4	1.288	0.807–2.057	0.289	F	18.4	0.298	0.734	0.959
Stage (I–II vs. III–IV)	3	0.818	0.327–2.044	0.667	F	5.3	0.348	1.000	0.648
OS	9	0.579	0.359–0.934	**0.025**	R	**84.9**	**0.000**	0.466	0.109
**Nestin Protein expression**									
Gender (male vs. female)	4	0.932	0.569–1.527	0.780	F	11.7	0.334	0.734	0.478
Age (≤ 60 vs. > 60 or ≤ 65 vs. > 65)	3	1.111	0.650–1.897	0.701	F	5.1	0.349	0.294	0.174
Smoking status (yes vs. no)	3	1.237	0.486–3.151	0.655	R	**60.7**	**0.078**	1.000	0.145
Histology (SCC vs. ADC)	4	2.378	0.420–13.462	0.327	R	**92.0**	**0.000**	0.734	0.542
Differentiation (well+moderate vs. poor)	3	2.671	0.170–41.861	0.484	R	**94.6**	**0.000**	1.000	0.335
Lymph node metastasis (N_1–3_ vs. N_0_)	2	**2.732**	**1.393–5.376**	**0.004**	F	0.0	0.694		
Stage (II–IV vs. I)	3	**1.996**	**1.157–3.445**	**0.013**	F	0.0	0.981	1.000	0.534
OS	5	**2.166**	**1.437–3.263**	**0.000**	R	**68.4**	**0.013**	0.806	0.534

### Nestin

**Figure 3:**
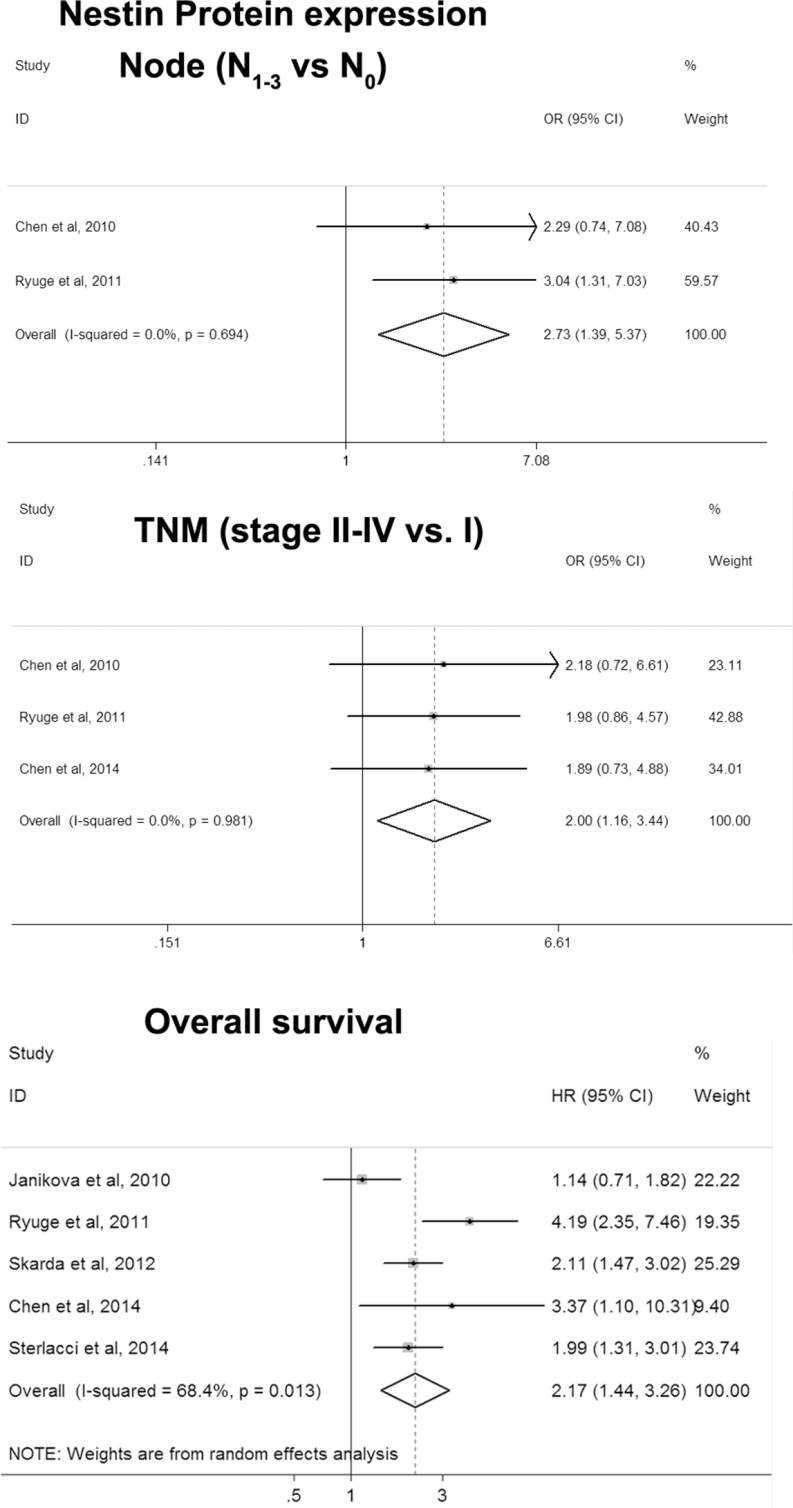
Forest plot for associations of *Nestin* with clinicopathological features and overall survival in NSCLC

There was no study reporting *Nestin* DNA amplification in NSCLC and then only *Nestin* protein expression was analyzed. The pooling analyses revealed significant associations of *Nestin* protein expression with lymph node matastasis (OR = 2.732, 95% CI = 1.393– 5.376, *P* = 0.004), stage (OR = 1.996, 95% CI = 1.157–3.445, *P* = 0.013), and OS (HR = 2.166, 95% CI = 1.437–3.263, *P* = 0.000) (Figure 3 and Table 2).

### Heterogeneity and sensitivity analysis

### SOX2

The heterogeneity and sensitivity were analyzed by subgroup analysis according to ethnicity, histology, and sample size or excluding single individual study. The results indicated that the heterogeneity existed in evaluating the associations of *SOX2* DNA amplification with gender (*I*^2^ = 55.6%), smoking status (*I*^2^ = 62.2%), histology (I^2^ = 89.7%), and differentiation (poor vs. well+moderate, *I*^2^ = 68.2%) (Table 2). For gender, heterogeneity decreased to 31.4% after excluding Wilbertz et al's study and the pooling OR was not influenced. For smoking status, after grouping by China and non-China studies, heterogeneity of both subgroups decreased to 0% and the associations were still significant. For histology, the heterogeneity could not be removed by subgroup analyses or excluding single individual study. For differentiation, the heterogeneity deceased (*I*^2^ = 19.3%) by excluding the study of Zhang et al. 2015 and the pooling OR was not influenced. Meanwhile, there were significant heterogeneity in assessment of the associations of *SOX2* protein expression with gender (*I*^2^ = 55.1%), age (*I*^2^ = 90.3%), smoking status (*I*^2^ = 58.9%), histology (*I*^2^ = 68.7%), and OS (*I*^2^ = 84.9%). The heterogeneity deceased significantly when deleting single individual studies of Chou et al. 2013 (gender, *I*^2^ = 38.7%), Chen et al. 2012 (age, *I*^2^ = 15.0%), Zheng et al. 2015 (smoking status, *I*^2^ = 0%), and Li et al. 2012 (histology, *I*^2^ = 34.5%), respectively. And the pooled ORs were not influenced, suggesting the results were stable. For OS, the heterogeneity still existed when excluding single individual study one by one. In subgroup analysis stratified by histology (SCC, *n* = 4; ADC, *n* = 1; and SCC/ADC, *n* = 4), heterogeneity was 29.7% in SCC, and 90.3% in SCC/ADC. And the association was significant in SCC but not SCC/ADC.

### Nestin

As shown in Table 2, there was heterogeneity in assessment of the associations of *Nestin* protein expression with smoking status (*I*^2^ = 60.7%), histology (*I*^2^ = 92.0%), differentiation (*I*^2^ = 94.6%), and OS (I^2^ = 68.4%). When the studies of Chen et al. 2010, Ryuge et al. 2011, and Janikova et al. 2012 were excluded, respectively, the heterogeneity significantly decreased (smoking status, *I*^2^ = 0%; differentiation, *I*^2^ = 0%; and OS, *I*^2^ = 48.2%) and the pooling ORs were not influenced except differentiation. As for histology, subgroup analysis suggested that the heterogeneity among the studies performed in China was decreased (*I*^2^ = 0%) and a significant association was presented. But the heterogeneity still existed in Japan group (*I*^2^ = 95.5%).

### Publication bias

**Figure 4:**
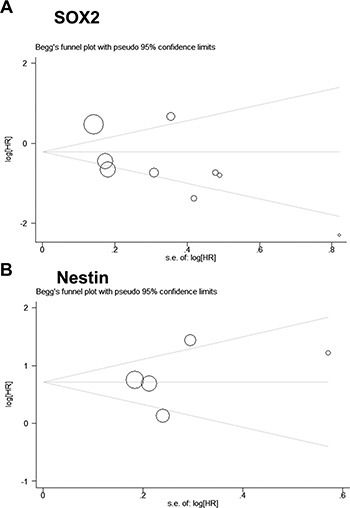
Begg's funnel plot for publication bias analysis

Furthermore, publication bias was also assessed by Begg's test and Egger's test. Symmetrical Begg's funnel plots and Egger's test results revealed no publication bias in all comparisons (Figure 4 and Table 2).

## DISCUSSION

A number of studies have been performed to explore the associations of cancer cell stem cell markers, such as *SOX2* and *Nestin*, with clinical parameters and prognosis in various types of cancers including NSCLC. However, the results in the studies were not consistent.

Up to now, there were two meta-analyses tying to investigate the associations of *SOX2* with clinicopathological features and/or overall survival in NSCLC [17, 57]. Chen et al. [17] only searched relevant studies in PubMed, up to May 2013 and included eight studies. Shao et al. [57] pooled seven studies published from 2010 to 2013. Neither of the studies distinguished *SOX2* DNA amplification, mRNA expression, and protein expression. In the present study, we analyzed *SOX2* DNA amplification and protein expression, respectively, unlike with the above reports. We searched in more English database and included more articles than the previous meta-analysis (19 vs. 8 and 7) although we did not combined the mRNA related studies. Pooling analyses suggested that both of the DNA amplification and the protein expression of *SOX2* were associated with smoking status, histology, and OS. In addition, *SOX2* DNA amplification was also associated with gender and differentiation. The discrepancy between DNA amplification and protein expression might be caused by the heterogeneity among studies and the inconsistency between amplification and protein expression. More studies examining amplification and protein expression of *SOX2* at the same time should be performed to confirm the conclusions. For *Nestin*, there was only one meta-analysis examining the associations of *Nestin* protein expression with TNM in regardless of cancer types. And the authors found that *Nestin* was positively associated with cancer stage and lymph node [58]. In the present meta-analysis, we summarized the associations of *Nestin* with clinicopathological features and OS in a single type of cancer, NSCLC. The pooling analyses suggested that high/positive *Nestin* was an indicator of poor prognosis in NSCLC, not as well as *SOX2*, which was a favorable factor for OS in NSCLC. This might bring us confusion when understanding the role of the two genes in molecular pathogenesis of NSCLC. Because both of them were cancer stem cell markers and mechanisms studies suggested that they all had proliferative and anti-apoptotic effects *in vitro* and animal model. Combined the results of the previous reports and the present meta-analysis, we proposed a mechanism model that *SOX2* was an oncogene and promoted tumorigenesis. Meanwhile, the tumors with *SOX2* up-regulation might exhibit a clearer squamous cell differentiation and were associated with better prognosis.

Although we pooled all the potential studies according to the inclusion and exclusion criteria, some limitations existed. Firstly, the number and sample size of *Nestin* related studies were small. Secondly, the studies of the subgroup of ADC for *SOX2* were rare. As the original studies suggested that *SOX2* was more frequently upregulated in SCC than ADC, the predictive role of *SOX2* in SCC and ADC might be not consistent. Then the impact of *SOX2* on prognosis in SCC and ADC should be compared in more studies with larger sample size.

In summary, we got a comprehensive result from the current meta-analysis that *SOX2* DNA amplification and protein expression were associated with smoking status and histology, and were favorable for prognosis in NSCLC. And *Nestin* was associated with cancer stage, lymph node, and poor outcome.

## MATERIALS AND METHODS

### Publication search

A systematic search was performed in four English databases (PubMed, EMBASE, OVID, and Web of science) for published articles on the associations of *SOX2* and *Nestin* with clinical features and/or overall survival (OS) in NSCLC up to November 10, 2015. The following keywords were used: “lung OR pulmonary”, “cancer OR tumor OR carcinoma”, and “*Nestin* OR Sex determining region Y box-2 OR SRY box-2 OR *SOX2*”. Two independent investigators screened the retrieved documents by reviewing the article titles, abstracts, or full texts according to the inclusion and exclusion criteria. The review articles and the references of selected articles were also screened to identify additional eligible studies.

### Inclusion and exclusion criteria

Inclusion criteria: (1) the histologic type of the tumors was NSCLC and if one study containing multiple types of lung cancer, only the data related to NSCLC was included; (2) evaluating the associations of *SOX2* and *Nestin* DNA amplification and/or protein expression with clinicopathological features and OS; (3) peer reviewed papers that have been published as full texts; (4) the language was limited as English; (5) if mutiple studies contained overlap or duplicated data, only the study with larger sample size was included. Exclusion criteria: (1) the frequency of patients with positive/negative/high/low DNA amplification and protein expression was not specific to clinicopathological features; (2) study with insufficient data; (3) abstracts, letters, or review articles.

### Data extraction

Two independent investigators collected related data carefully and the following characteristics were extracted from included studies: first author name, year of publication, country, ethnicity, patient number, gender, age, protein expression/amplification, method, cut-off value, smoking status, histologic type, differentiation, lymph node metastasis, stage, and OS.

### Statistical analysis

All the statistical analyses were carried out with the software Stata 12.0 (StataCorp, College Station, TX, USA). The crude odds ratios (ORs) with 95% confidence intervals (95% CIs) were calculated to estimate the associations of DNA amplification and protein expression of *SOX2* and *Nestin* with clinicopathological features of NSCLC. The crude hazard ratios (HRs) with 95%CIs were used to assess their clinical significance in predicting prognosis of NSCLC. The statistical significant level was determined by *Z*-test with *P* value less than 0.05. If the prognosis was only presented by a Kaplan-Meier plot curve, HR and its 95%CI were calculated according to previous reports [59, 60]. Briefly, the KM plot curves were read by Engauge Digitizer version 2.11 and HR was estimated by the calculation spreadsheet. The spreadsheet could be freely downloaded from http://www.trialsjournal.com/content/supplementary/1745-6215-8-16-s1.xls. Inconsistency was solved by discussion. The heterogeneity among studies was explored using the chi-square based on Q statistic test. If P > 0.1 or *I*^2^ < 50%, fixed effects model was used to calculate the pooled OR/HR. Otherwise, random-effects model was used [61]. Sensitivity analysis was also conducted to evaluate stabilities of pooling results by omitting studies that brought heterogeneity or publication bias. Potential publication bias was checked by Begg's funnel plots and Egger's test [62, 63].
